# In vivo Confocal Microscopy of Posner-Schlossman Syndrome: Comparison with herpes simplex keratitis, HLA-B27 anterior uveitis and acute attack of primary angle closure

**DOI:** 10.1038/s41598-017-10496-7

**Published:** 2017-08-29

**Authors:** Ying Hong, Miao Wang, Lingling Wu

**Affiliations:** 1 Department of Ophthalmology, Peking University Third Hospital Key Laboratory of Vision Loss and Restoration, Ministry of Education, No.49 North Garden Road, Haidian District, Beijing, China; 2 Department of Ophthalmology, Beijing Children’s Hospital, No.56 South Lishi Road, Xicheng District, Beijing, China

## Abstract

To investigate *in vivo* confocal microscopy (IVCM) findings in patients with Posner-Schlossman Syndrome (PSS), we compared the IVCM findings from the eyes of patients with: PSS (44 eyes); herpes simplex keratitis (HSK) (45 eyes); HLA-B27 anterior uveitis (B27AU) (45 eyes); and with acute attack of primary angle closure (aPAC) (43 eyes). The central Langerhans cells (LCs) grade at the level of corneal basal epithelial cells of the PSS group (2.33 ± 0.55) was similar to that of the HSK group (2.63 ± 0.67) (χ^2^ = −1.435, *P* = 0.174) but was significantly higher than those of the B27AU group (1.80 ± 0.79) (χ^2^ = 2.311, *P* = 0.023) and the aPAC group (1.75 ± 0.46) (χ^2^ = 2.701, *P* = 0.022). The keratocyte activation grade of the PSS group (1.55 ± 0.76) was similar to that of the HSK group (1.65 ± 0.81) (χ^2^ = 1.104, *P* = 0.675) but was significantly higher than those of the B27AU group (1.00 ± 0.71) (χ^2^ = 2.364, *P* = 0.025) and aPAC group (1.75 ± 0.46) (χ^2^ = 2.532, *P* = 0.027). The LCs and keratocyte activation grades observed by IVCM in patients with PSS were higher than those in patients with B27AU and with aPAC, but they were similar to those in patients with HSK. This implies that PSS might be related to viral infection.

## Introduction

Glaucomatocyclitis crisis, also known as Posner-Schlossman Syndrome (PSS), is an uncommon form of cyclitis with recurrent intraocular pressure (IOP) elevation^1^. It is identified as a unilateral condition of recurrent mild cyclitis with a few keratic precipitates (KP). The IOP may increase to 40–70 mmHg at the stage of attack^1–4^. PSS is typically seen in the age group of 20–50-year-olds^1, 4^, but there are reports that it may also affect children and the elderly^5^. It is important to distinguish PSS with acute attack of primary angle closure (aPAC) from iridocyclitis because they may both present as a unilateral hypertensive attack^4, 6, 7^. The cause of PSS remains unclear, but the most possible cause is a viral infection, especially cytomegalovirus^8–12^. Teoh SB *et al*. found cytomegalovirus in the aqueous humour of immunocompetent PSS patients^8^. New evidence that further shows PSS to have a relationship with a viral infection would be of great importance for the diagnosis and treatment of PSS.

*In vivo* confocal microscopy (IVCM) is a noninvasive technique that can demonstrate anatomical details at the tissue level. IVCM allows for *in vivo* examination of the human cornea at all cellular levels by continuous confocal scanning^13^. Currently, it is widely used in the diagnosis of corneal diseases, such as viral keratitis, keratoconus and corneal dystrophy^14–18^. Its characteristics of high resolution (1 μm) and high magnification make it possible to observe the morphology of KP and corneal cells *in vivo* ^18–23^.

Recently, some studies mentioned the IVCM findings in eyes with viral keratitis^18, 19, 21, 23^, iridocyclitis^22^, and Fuchs’ uveitis syndrome^20^. However, as far as we know, the IVCM findings in PSS eyes are lacking.

Therefore, the purpose of this study is to identify the corneal morphology of PSS patients by IVCM and compare it with the corneal morphologies of patients with herpes simplex keratitis (HSK), HLA-B27 anterior uveitis (B27AU) and aPAC.

## Results

In total, there were 44 PSS patients enrolled, including 25 males and 19 females. Their ages ranged from 14 to 79 years, with an average age of 38.6 years. There were 30 patients (68%) whose ages were between 20 and 50 years. Forty-five patients with HSK, 47 with B27AU and 43 with aPAC were enrolled in the control groups at the same time.

The general information of the participants is shown in Table 1.

**Table 1:** The general information of the participants ($\bar{x}\pm s$).

Group	PSS (n = 44)	HSK (n = 45)	B27AU (n = 47)	aPAC (n = 43)	*F* value	*P* value
Age (yrs)	44.5 ± 18.4	52.0 ± 18.6	42.4 ± 14.7	57.4 ± 6.1	2.525	0.063
Gender (M/ F)	25/19	24/21	28/19	21/22	1.961	0.126
VA (LogMAR)	0.2 ± 0.2	0.3 ± 0.2	0.3 ± 0.3	0.4 ± 0.3	1.362	0.260
IOP (mmHg)	30.7 ± 14.4	14.5 ± 2.9	13.6 ± 4.1	18.6 ± 6.6	19.142	<0.001

PSS: Posner-Schlossman Syndrome; HSK: herpes simplex keratitis; B27AU: HLA-B27 anterior uveitis; aPAC: acute attack of primary angle closure.

There were no statistically significant differences between the age, gender ratio, and VA of patients in the PSS group and those of the patients in the other 3 groups (*F* = 2.525 and *P* = 0.063, *F* = 1.961 and *P* = 0.126, and *F* = 1.362 and *P* = 0.260, respectively). The IOP of the PSS group was much higher than that of the other 3 groups, and the *P* value of the differences in the IOP was less than 0.001 between the PSS group and the other 3 groups (*F* = 19.142, *P* < 0.001).

The IVCM findings of the LCs and keratocyte activation grades of the 4 groups are shown in Table 2.

**Table 2:** The IVCM findings of LCs and keratocyte activation grades of the 4 groups ($\bar{x}\pm s$).

Group	PSS (n = 44)	HSK (n = 45)	B27AU (n = 47)	aPAC (n = 43)	*F* value	*P* value
LCs	2.33 ± 0.55	2.63 ± 0.67	1.80 ± 0.79	1.75 ± 0.46	5.102	0.004
Keratocyte activation	1.55 ± 0.76	1.65 ± 0.81	1.00 ± 0.71	0.91 ± 0.70	4.126	0.010

PSS: Posner-Schlossman Syndrome; HSK: herpes simplex keratitis; B27AU: HLA-B27 anterior uveitis; aPAC: acute attack of primary angle closure.

**Figure 1:**
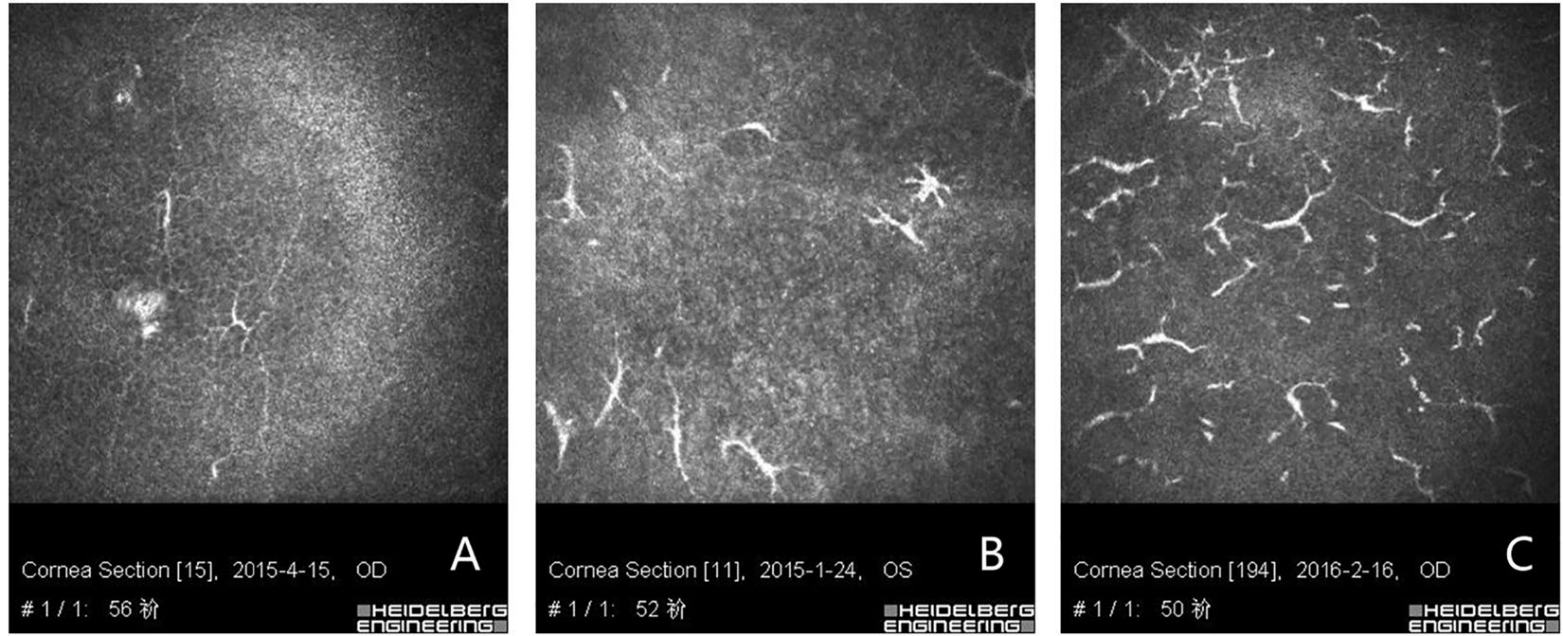
Langerhans cells (LCs) grade of eyes with Posner-Schlossman Syndrome. (**A**) Grade 1: 1 to 5 dendritic cells. (**B**) Grade 2: 6 to 20 dendritic cells. (**C**) Grade 3: >20 dendritic cells.

**Figure 2:**
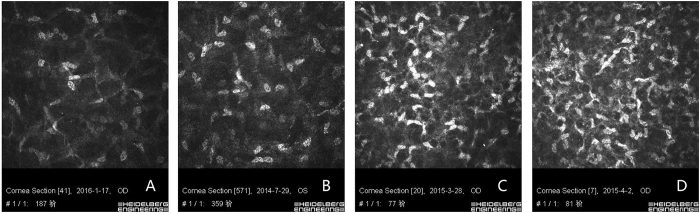
Keratocyte activation grade of a PSS patient. (**A**) Grade 0: 0 to 25% of keratocytes exhibit activation. (**B**) Grade 1: 25 to 50% of keratocytes exhibit activation. (**C**) Grade 2: 50 to 75% of keratocytes exhibit activation. (**D**) Grade 3: 75 to 100% of keratocytes exhibit activation.

Briefly, the LCs grade of the PSS group was similar to that of the HSK group (χ^2^ = −1.435, *P* = 0.174) but significantly higher than those of the B27AU group (χ^2^ = 2.311, *P* = 0.023) and aPAC group (χ^2^ = 2.701, *P* = 0.022) (Fig. 1). The keratocyte activation grade of the PSS group was similar to that of the HSK group (χ^2^ = 1.104, *P* = 0.675) but significantly higher than those of the B27AU group (χ^2^ = 2.364, *P* = 0.025) and aPAC group (χ^2^ = 2.532, *P* = 0.027) (Fig. 2).

**Figure 3:**
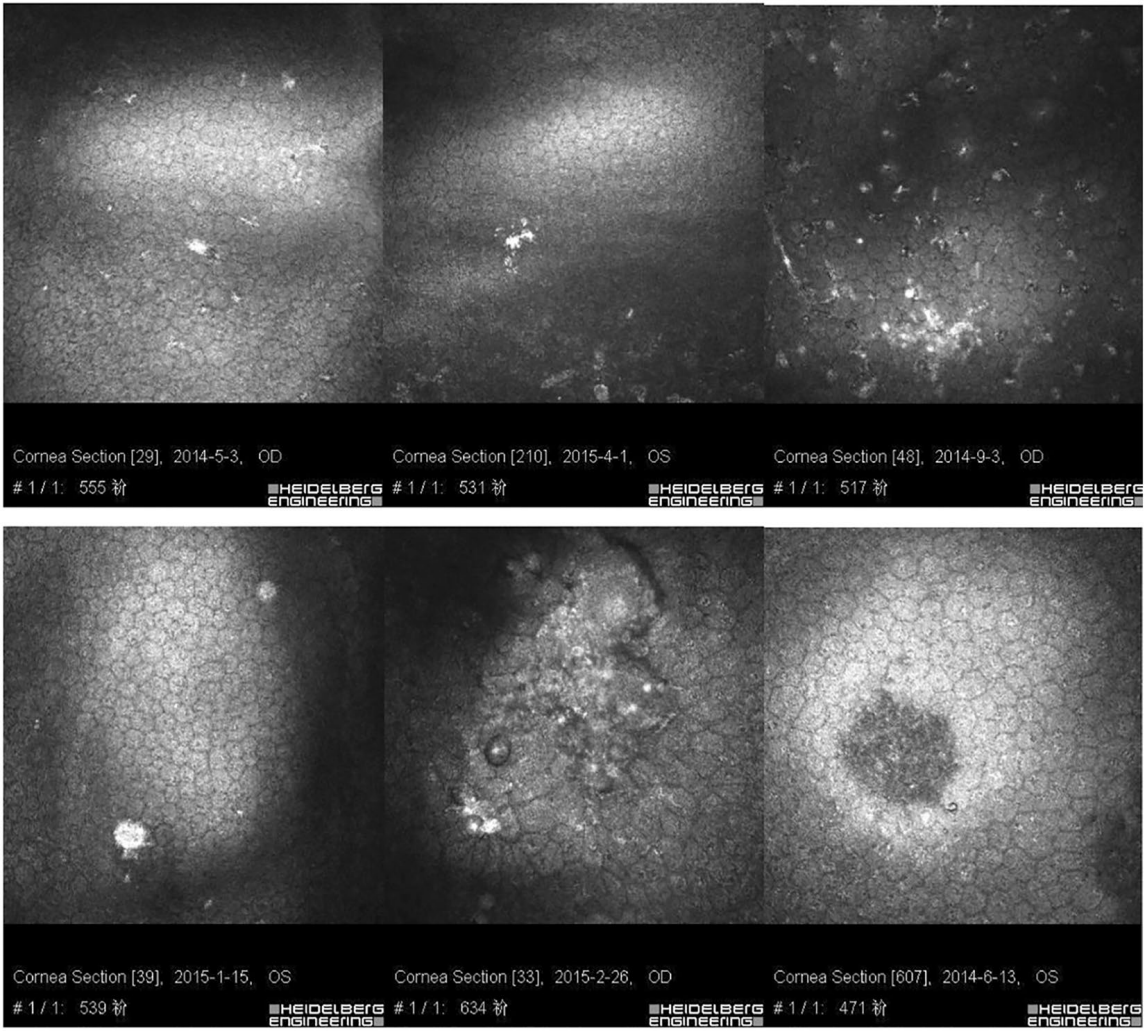
Six types of KPs in Posner-Schlossman Syndrome patients. (From upper left to lower right) type I: small and round KPs; type II: stipped KP; type III: dendritiform KP with threadlike extensions across the endothelium; type IV: large and smooth-rounded KP; type V: globular KP with large and harbouring multiple hyperreflective round inclusions in a conglomerate appearance; type VI: endothelial blebs observed as empty lacunae.

**Figure 4:**
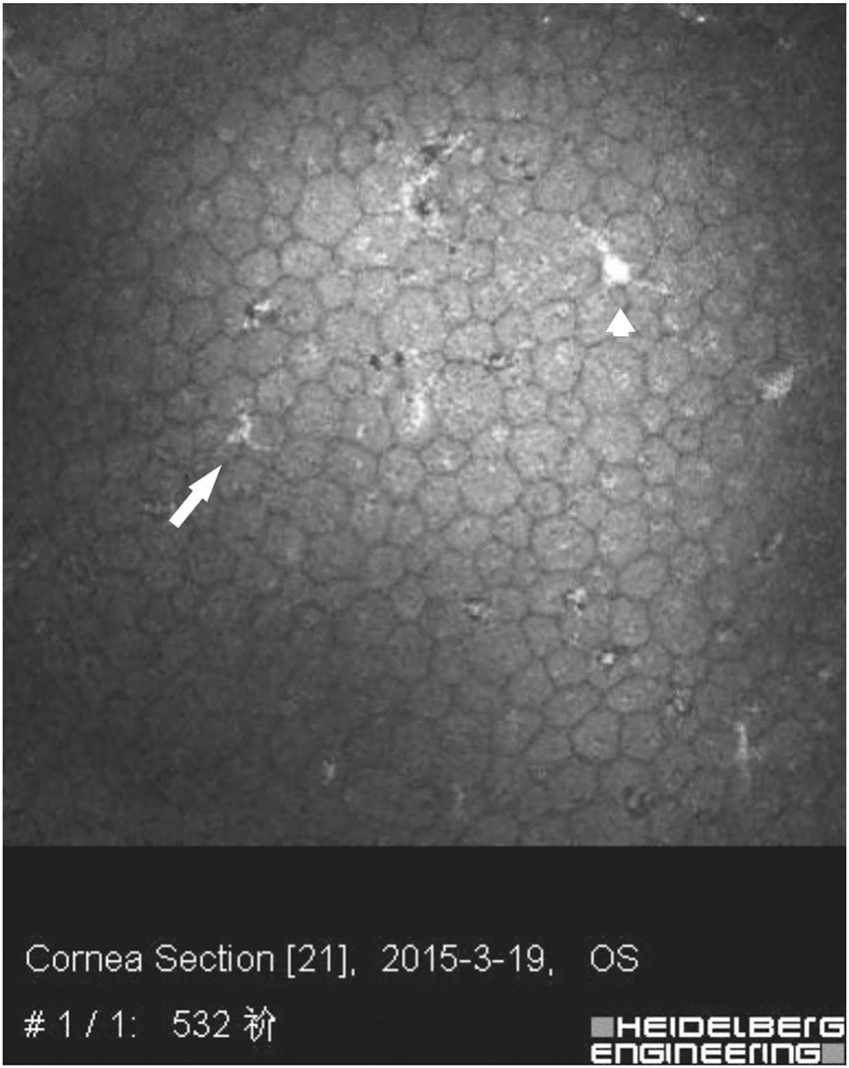
Two kinds of KP were observed in one PSS eye: type I (arrow) and type III (arrow head).

The morphology of the corneal endothelial cells was normal in the eyes of patients with PSS as shown in Fig. 3. However, different types of KPs were seen in 34 (77%) of the patients with PSS. Among them, 6 (18%) were type I, 7 (21%) were type II, 7 (21%) were type III, 2 (6%) were type IV, 7 (21%) were type V and 5 (15%) were type VI (Fig. 3). Additionally, in 4 patients (12%), more than 2 types of different KPs were observed (Fig. 4).

## Discussion

The current study showed that the LCs grade and keratocyte activation grade of eyes with PSS were similar to those of eyes with HSK and that IVCM detected different KPs in 77% of them. To our knowledge, this is the first study to evaluate IVCM images in PSS patients. Our results indicated that the inflammatory reaction of PSS affected the corneal stromal layer, which to our knowledge has not been recorded previously^1^.

PSS is characterized by an IOP elevation, corresponding corneal epithelial oedema and anterior chamber inflammation. Eyes with aPAC, HSK and B27AU have been used as control groups because each of them has one similar typical characteristic of PSS. The IOP would always be elevated in eyes with aPAC, while corneal inflammatory oedema often exists in eyes with HSK, even though it is usually not of the corneal epithelial oedema type, and anterior chamber inflammation occurs in eyes with B27AU.

The appearance of LCs at the corneal level indicates local inflammation. The LCs grade in eyes with PSS was similar to that in those with HSK and much higher than those in eyes with B27AU and aPAC. LCs might be seen as being from three different morphologies: those without dendrite-like protrusions, those with short dendrite-like protrusions and those with long dendrite-like protrusions at the corneal epithelial and anterior stromal levels in about one-third of normal persons^24, 25^. In normal eyes, the LCs are usually immature without dendrite-like protrusions forming in the central cornea and in dendrite-like protrusions forming in the peripheral cornea. Under the stimulation of an inflammatory factor or a chemotactic factor, immature LCs at the corneal epithelial level could be activated to multiple cells with dendrite-like protrusions as mature LCs with the motility of moving vertically or along the nerves in the cornea^26^. The degree of activation of the LCs was in accordance with inflammation^13, 24, 25^. That might explain why LCs were observed by IVCM in the corneal basal epithelial cell layer of eyes with PSS in our study. The LCs observed in the current study were cells with multiple dendrite-like protrusions, which meant that they were fully activated.

In the current study, the keratocyte activation grade in eyes with PSS was similar to that of eyes with HSK and much higher than that of eyes with B27AU and that of eyes with aPAC. Keratocytes originate from the mesenchyme of the neural crest in the embryonic period. Being a type of fibroblast, they are usually very stable and seldom undergo mitosis under a physiological state. Keratocytes connect with nearby cells by gap junctions and form the neuronal network appearance^27^. When certain keratocytes were damaged, other cells that are surrounding them would be activated by gap junctions and would secrete an extracellular matrix. The activated keratocytes may be created from mitosis and migration. Therefore, keratocyte activation usually indicates inflammation of the corneal stroma. In addition, inflammation can be clinically observed in the anterior chamber, and the IVCM results in our study showed that the inflammatory reaction in eyes with PSS was also in seen in the corneal stroma, which was similar to that in the eyes with HSK.

In our study, LC maturation and keratocyte activation were observed in eyes with an elevated IOP. Ocular inflammation in aPAC patients due to an elevated IOP may destroy the aqueous humour-blood barrier. Elevated concentrations of interleukins, granulocyte colony-stimulating factor and vascular endothelial growth factor were shown in the aPAC patients^28^. The concentrations of these cytokines might parallel the level of the IOP^29^. Although the symptoms of PSS patients, such as an elevated IOP and ocular pain, are sometimes easily confused with those of aPAC patients, there was significant difference in both the grades of LCs and keratocyte activation between the two groups. For the PSS group, the grades of LCs and keratocyte activation were much higher than those of the aPAC group, in which only an elevated IOP could not be fully explained.

Noninfectious inflammation may also cause keratocyte activation and LCs maturation^22^. As a kind of noninfectious uveitis, B27AU is a kind of autoimmune disease. The inflammation is usually severe in the parts that are full of vessels, such as the iris and the ciliary body, but it is less severe in the cornea because vessels are rarely found.. In the current study, the grades of LCs and keratocyte activation were much higher in the PSS group than in the B27AU group, which could not fully be explained by noninfectious anterior chamber inflammation.

Currently, the cause of PSS remains unclear. Recent studies have demonstrated that it is related to a viral infection^8–12^. Furthermore, the latest study showed that it might be due to the immunoreaction in which a viral infection is triggered^7^. The reason why patients with HSK were chosen as one of the control groups was because a large number of LCs could be seen in their corneas^14–16^. The mean grades of LCs and keratocyte activation in the PSS group were similar to those in the HSK group. The above findings indicated that the IVCM findings in eyes with PSS were much more similar to those in eyes with HSK compared to those in eyes with aPAC and in eyes with B27AU. Such IVCM findings also implied that PSS might have a relationship with a viral infection from a morphology perspective.

We also found that the KPs in the eyes with PSS had multiple morphologies under the IVCM. Though corneal endothelial cells were almost normal, up to 77% of the PSS eyes had KPs, and 12% of them had more than one type of KP. Though KPs in uveitis eyes have been reported^21, 22^, there have been a very limited number of studies about KPs in PSS patients. The only reports that we can find are by Pilli CT *et al*., who observed single and globular KPs in PSS eyes using a Tomey-1100 wide field contact specular microscope but not by IVCM^30^. The current IVCM study found 6 types of KPs in eyes with PSS, which was consistent with those found in eyes with uveitis^21^.

The current study has some limitations. First, the number of patients was limited due to IVCM being a kind of contact examination, and the PSS patients should be at an acute episode. Second, there might be unnoticed changes because the range of IVCM is limited. Third, the current research lacked the longitudinal results of the PSS patients. We are currently following-up the patients and will do further study and analyses.

In conclusion, the current study showed the IVCM findings of PSS patients and compared them with those of patients with HSK, patients with B27AU and patients with aPAC. There were LCs and keratocyte activation in many eyes with PSS, which was similar to what was observed in eyes with HSK. The IVCM findings also demonstrated that different types of KPs were present in PSS patients. Such findings indicate that the corneal activation of the PSS patients might have a relationship with a viral infection.

## Methods

### Participants

This prospective comparative study was conducted at a single university-based hospital, the Peking University Third Hospital, from May 2014 to May 2016. Written informed consent was obtained from all of the subjects, and the study was carried out with the approval of the Institutional Review Board of the Peking University Third Hospital. The study adhered to the tenets of the most recent revision of the Declaration of Helsinki. Consecutive patients clinically diagnosed as having PSS during that time period were included in the study. General information and a detailed ocular examination included vision acuity (VA), intraocular pressure (IOP), slit lamp photography and IVCM, and especially LCs grade, keratocyte activation grade and the morphology of KP. The control groups consisted of patients with HSK, patients with B27AU and patients with aPAC.

The diagnosis of PSS was established on the detection of the following clinical features by two senior glaucoma specialists: (a) recurrent episodes of mild iritis associated with elevated IOPs (>21 mmHg), with or without diffuse corneal epithelial oedema of the cornea, and a few KPs, (b) a normal IOP between attacks and normal and open angles^10^, (c) a unilateral attack, and (d) immunocompetency from the patient^7^. All of the patients were without peripheral iris anterior synechiae and posterior synechiae.

The diagnosis of HSK was done according to the clinical criteria of Liesegang and Schwartz: (a) with or without a previous history of viral keratitis, (b) a unilateral attack, (c) typical clinical keratitis findings, including diffuse corneal oedema or disciform stromal oedema, KP, and/or anterior chamber inflammation at varying degrees^31, 32^.

The diagnosis of B27AU included the following: (a) a documented presence of recurrent anterior uveitis with chronic low back stiffness of at least 3 months’ duration, (b) radiologic evidence of sacroiliitis on magnetic resonance imaging, (c) HLA-B27 positivity, and (d) an active intraocular inflammation in patients at the time of evaluation, including but not limited to red eye, pain, photophobia, non-granulomatous uveitis, KP, and the presence of cells or proteins in the aqueous humour^21, 33^.

All of the patients with aPAC who were included in the current study were experiencing an acute attack at the time of examination or within 1 week of examination. aPAC was defined according to the following criteria: (a) the presence of at least 2 of the following symptoms: ocular or peri-ocular pain, nausea, and/or vomiting, and an antecedent history of intermittent blurring of vision with halos; (b) presenting an IOP of at least 22 mmHg (as measured by Goldmann applanation tonometry, GAT); (c) the presence of at least 3 of the following signs of conjunctival injection: corneal epithelial oedema, a mid-dilated unreactive pupil, and a shallow anterior chamber; and (d) the presence of an occluded angle in the affected eye^28, 34–36^. If the corneal oedema was too severe to be imaged by IVCM, the examination would be performed again several days after medical treatment.

### *In vivo* confocal microscopy

**Figure 5:**
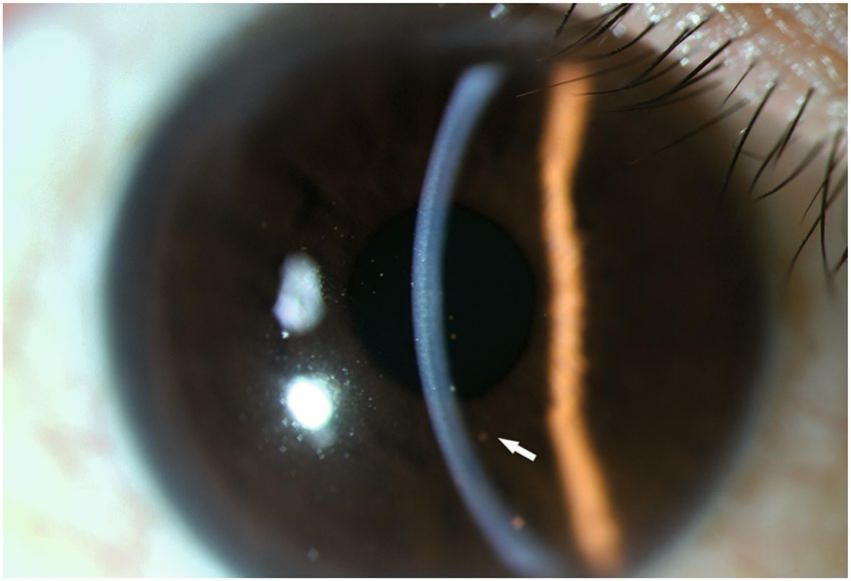
Slit lamp photo of a Posner-Schlossman Syndrome patient. The cornea is clear with several mutton-fat KPs (arrow) at the corneal endothelial layer.

Anterior segment photos were recorded for all of the patients (Fig. 5). All of the patients were examined by a digital corneal confocal laser-scanning microscope (*in vivo* confocal microscopy, Rostock Cornea Module, HRT II RCM Heidelberg Engineering Inc., Heidelberg, Germany) that was equipped with the in-built software Heidelberg Eye Explorer version 1.5.10.0. The IVCM uses a helium neon diode laser source with a wavelength of 670 nm. The two-dimensional images captured by the IVCM have a definition of 384 × 384 pixels over an area of 400 × 400 μm, with a lateral spatial resolution of 0.5 μm, a mean magnification of ×800 and a depth resolution of 1 μm^26^.

The patients were seated comfortably at the IVCM, with their chin resting on a standard slit lamp frame. On “section” mode, one experienced technician captured images. The PSS patients, HSK patients and B27AU patients were examined in the primary position and/or in elevation, depending on the location of the KP, while the aPAC patients were examined in the primary position. The images from the corneal epithelial level to the endothelial level were captured and analysed in a masked fashion by two cornea specialists and one glaucoma specialist.

### Image analysis

Langerhans Cells: The LCs were hyperreflective corpuscular particles with or without dendrite-like protrusions situated at the level of basal epithelial cells and the sub-basal nerve plexus. LCs with processes indicated that they were mature ones^26^ Because the appearance of mature LCs at the central corneal basal epithelium indicates inflammation, in our study, we evaluated the eyes with mature LCs at the level of the basal epithelial cell layer and sub-basal nerve plexus (approximately 40–60 μm from the corneal surface). The LCs were classified from grade 0 to grade 3 based on published articles^37^: grade 0: no dendritic cells; grade 1:1 to 5 dendritic cells; grade 2:6 to 20 dendritic cells; grade 3: >20 dendritic cells. The grading was based on the maximum number of cells that were counted in a 400 × 400 μm frame^37^.

Keratocyte activation: In the normal corneal stromal layer, reflections from keratocyte nuclei were seen against a darker background. The nuclei were separated by an amorphous ground substance. The cytoplasm, cell boundaries, and collagen lamellae were not visible^27^. However, the activated keratocytes were swollen, with an increased reflection and visible cytoplasmic processes^37^. Activated keratocytes were classified from grade 0 to grade 3 based on published articles^37^: grade 0: 0 to 25% of keratocytes exhibit activation; grade 1:25 to 50% of keratocytes exhibit activation; grade 2:50 to 75% of keratocytes exhibit activation; grade 3:75 to 100% of keratocytes exhibit activation. The grading was evaluated at a depth of 100 μm to minimize interference from subepithelial and/or anterior stromal hyperreflectivities^37^.

KP: Based on a previous study, the KPs were classified in six groups as follows: type I, small and round KPs; type II, stipped KPs; type III, dendritiform KPs with threadlike extensions across the endothelium; type IV, large and smooth-rounded KPs; type V, globular KPs with large and multiple hyperreflective round inclusions in a conglomerate appearance; and type VI, endothelial blebs observed as empty lacunae^21^.

### Statistical analysis

The statistical analyses were performed using SPSS 19.0(SPSS, Inc. Chicago, IL). For continuous variables, such as age, VA and IOP, the data were calculated as the $\bar{x}\pm s$ (Mean ± Standard Deviation) for each group and compared by one-way ANOVA, while further analyses between two groups were compared using the LSD-t test when they were normally distributed and using the homogeneity of variance. For categorical variables, such as LCs and keratocyte activation grades in each group, the frequency distributions were calculated and compared by the χ2 test. A P-value of less than 0.05 was considered statistically significant.
